# miR155 deficiency aggravates high‐fat diet‐induced adipose tissue fibrosis in male mice

**DOI:** 10.14814/phy2.13412

**Published:** 2017-09-26

**Authors:** Kandy T. Velázquez, Reilly T. Enos, Meredith S. Carson, Taryn L. Cranford, Jackie E. Bader, Alexander T. Sougiannis, Cara Pritchett, Daping Fan, James A. Carson, E. Angela Murphy

**Affiliations:** ^1^ Department of Pathology, Microbiology, and Immunology School of Medicine University of South Carolina Columbia South Carolina; ^2^ Department of Cell Biology and Anatomy School of Medicine University of South Carolina Columbia South Carolina; ^3^ Department of Exercise Science University of South Carolina Columbia South Carolina

**Keywords:** Adipose tissue fibrosis, high‐fat diet, inflammation, MicroRNA 155, obesity

## Abstract

Noncoding RNAs are emerging as regulators of inflammatory and metabolic processes. There is evidence to suggest that miRNA155 (miR155) may be linked to inflammation and processes associated with adipogenesis. We examined the impact of global miRNA‐155 deletion (miR155^−/−^) on the development of high‐fat diet (HFD)‐induced obesity. We hypothesized that loss of miR155 would decrease adipose tissue inflammation and improve the metabolic profile following HFD feedings. Beginning at 4–5 weeks of age, male miR155^−/−^ and wild‐type (WT) mice (*n* = 13–14) on a C57BL/6 background were fed either a HFD or low‐fat diet for 20 weeks. Body weight was monitored throughout the study. Baseline and terminal body composition was assessed by DEXA analysis. Adipose tissue mRNA expression (RT‐qPCR) of macrophage markers (F4/80, CD11c, and CD206) and inflammatory mediators (MCP‐1 and TNF‐*α*) as well as adiponectin were measured along with activation of NF*κ*B‐p65 and JNK and PPAR‐*γ*. Adipose tissue fibrosis was assessed by picrosirius red staining and western blot analysis of Collagen I, III, and VI. Glucose metabolism and insulin resistance were assessed by Homeostatic Model Assessment – Insulin Resistance (HOMA‐IR), and a glucose tolerance test. Compared to WT HFD mice, miR155^−/−^ HFD mice displayed similar body weights, yet reduced visceral adipose tissue accumulation. However, miR155^−/−^ HFD displayed exacerbated adipose tissue fibrosis and decreased PPAR‐*γ* protein content. The loss of miR155 did not affect adipose tissue inflammation or glucose metabolism. In conclusion, miR155 deletion did not attenuate the development of the obese phenotype, but adipose tissue fibrosis was exacerbated, possibly through changes to adipogenic processes.

## Introduction

Lack of physical activity and excessive energy consumption has resulted in a global obesity epidemic, which has been linked to increased risks of mortality, morbidity, and economic costs (Nguyen and El‐Serag 2010). The obese phenotype embodies a chronic state of low‐grade inflammation and metabolic disturbances, two underlying characteristics believed to account for obesity's detrimental effect on health. Thus, developing therapies and unraveling key molecules to combat obesity‐associated inflammation and metabolic disorders are of significant public health importance.

One of the cell populations believed to be most responsible for the chronic inflammation and metabolic disturbances characteristic of the obese state is macrophages (Boutens and Stienstra 2016). In the obese condition, proinflammatory, M1, macrophages infiltrate adipose tissue, leading to a highly inflammatory environment(Lumeng et al. 2007b). M2, resident macrophages, on the other hand, serve as anti‐inflammatory mediators. As obesity progresses, an imbalance of macrophage subtype develops, with M1 macrophages serving as the dominant macrophage phenotype present, resulting in a net, proinflammatory environment, which has been shown to negatively affect metabolic processes (Lumeng et al. 2007a,c, 2008; Lee et al. 2011; Boutens and Stienstra 2016). Therefore, understanding the integral regulators involved in macrophage recruitment and polarization are of critical importance to treat the underlying inflammatory condition typical of the obese state.

Significant attention has recently been given to microRNAs (miRNAs), as these small noncoding RNAs are known to be involved in the inflammatory and dysfunctional metabolic processes linked to obesity (Hilton et al. 2013; Abente et al. 2016). One such miRNA, miRNA155 (miR155), plays an integral role in adipocyte processes, including adipogenesis and browning of adipocytes (Liu et al. 2011; Chen et al. 2013). Additionally, miR‐155 has been shown to promote macrophage polarization to an M1 phenotype, is more highly expressed in M1 macrophages compared to M2 macrophages, and plays a key role in pro‐inflammatory processes (Zhang et al. 2013, Essandoh et al., 2016). However, the influence that miR155 has on obesity development, including in vivo macrophage behavior, adipose tissue physiology, and glucose metabolism remains poorly understood and published findings are equivocal. For example, miR155 deletion has been shown to prevent diet‐induced obesity in a sex‐dependent manner (Gaudet et al. 2016), while a recently published study found no such effect (Virtue et al. 2016). Additionally, a study by Csaket al. (2015) indicated that miR155 deficiency attenuated nonalcoholic fatty‐liver disease (NAFLD), a condition typically associated with obesity, yet in another model of NAFLD, deletion of miR155 augmented NAFLD development (Miller et al. 2013).

The purpose of our investigation was to examine miR155 regulation of macrophage phenotype, adipose tissue inflammation, and metabolism in a mouse model of high‐fat diet (HFD)‐induced obesity. Using male miR155^−/−^ mice, we hypothesized that loss of miR155 would reduce M1 macrophage accumulation in adipose tissue, resulting in decreased adipose tissue inflammation and an improved metabolic profile compared to wild‐type (WT) mice in an HFD‐induced obesity setting.

## Methods

### Animals

Male WT C57BL/6 and miR155^−/−^ mice on a C57BL/6 background were originally purchased from the Jackson Laboratories (Bar Harbor, ME) and were subsequently bred as separate colonies (e.g., miR155^−/−^ x miR155^−/−^ and WT x WT) at the Animal Resources Facility at the University of South Carolina. Mice were weaned and genotyped at 3 weeks of age. miR155 deletion was confirmed at sacrifice via qRT‐PCR. Mice were housed up to 5 per cage in a 12:12 h dark‐light cycle in a low stress environment (22°C, 50% humidity, low noise) and given food and water ad libitum. Principles of laboratory animal care were followed, and the Institutional Animal Care and Usage Committee of the University of South Carolina approved all experiments.

### Diets

At 4–5 weeks of age, WT and miR155^−/−^ mice were randomly assigned to one of two diets for a total of 20 weeks: a purified HFD (40% of total kcals from fat) designed to mimic the standard American diet (Enos et al. 2013, 2014b) or the AIN‐76A, purified low‐fat diet (LFD) (BioServ, Frenchtown, NJ).

### Body weights, food intake, and body composition

Body weight and food intake were monitored weekly. Body composition was assessed at baseline and after 20 weeks of dietary treatment via dual‐energy X‐ray absorptiometry (DEXA) (Lunar PIXImus, Madison, WI).

### Metabolism

After 19 weeks of dietary treatment, blood samples were collected from the tip of the tail after a five‐hour fast. Blood glucose concentrations were determined in whole blood, using a glucometer (Bayer Contour, Michawaka, IN). Collected blood was centrifuged at 10,600 *g* for 10 min at 4°C. Plasma was aliquoted and stored at −80°C until analysis. Plasma insulin concentrations were performed according to the manufacturer's instructions Mercodia mouse insulin ELISA kit (Mercodia Inc., Winston Salem, NC). Insulin resistance was estimated by the Homeostatic Model Assessment Index – Insulin Resistance (HOMA‐IR) according to the following formula: insulin resistance index = fasting insulin (*μ*U/ml) x fasting glucose (mmol/l)/22.5. Glucose tolerance tests (GTTs) were performed after 19 weeks of dietary treatment. Briefly, mice were fasted for 5 h and glucose was administered (IP) at a dose of 2 g/kg lean mass. Blood glucose concentrations (tail sampling) were measured intermittently over a two‐hour period (0, 15, 30, 60, 90, and 120 min), using a glucometer (Bayer Contour, Michawaka, IN). Area under the curve (AUC) was calculated, using the trapezoidal rule.

### Tissue collection

After 20 weeks of dietary treatment, mice were sacrificed via isoflurane inhalation for tissue collection. Tissues were removed, weighed, and immediately snap‐frozen in liquid nitrogen and stored at −80°C or fixed in 4% paraformaldehyde until analysis.

### H&E and picrosirius red staining and adipocyte size

H&E and Picrosirius Red staining (Picrosirius Red Staining kit (Abcam, Cambridge, UK)) were performed on epididymal adipose tissue. Representative images were taken at 20× magnification on a Nikon E600. For adipocyte size, adipocytes from 20× images were traced and analyzed, using the MRI_Adipocytes_Tool macro for Image J software (NCBI)(Parlee et al. 2014).

### Quantitative real‐time RT‐PCR

In order to confirm miR‐155 deficiency in miR‐155^−/−^ mice, a small portion of the liver from each mouse was utilized to extract miRNA, using the miRNeasy mini Kit (Qiagen, Valencia, CA) following the manufacturer's instructions. RNA was isolated from epididymal adipose tissue, using RNeasy Lipid Tissue Mini Kit (Qiagen, Valencia, CA). TaqMan reverse transcription reagents and MicroRNA and mRNA assays (Applied Biosystems, Foster City, CA) were used to reverse transcribe and to analyze the expression of the following targets: miR‐155 and sno202 and F4/80, CD11c, CD206, MCP‐1, and TNF*α*, respectively. Various genes were examined as potential endogenous controls for mRNA assays, including 18s rRNA, RPLP2, HPRT, and HMBS. HMBS was found to be the most stable and thus was used as the reference gene for mRNA samples whereas sno202 was utilized as the reference gene for miR‐155 analysis. Gene expression quantification was calculated, using the ΔΔCT method.

### Western blot analysis

Briefly, epididymal adipose tissue was homogenized in Mueller Buffer containing a cocktail protease inhibitor (Sigma Aldrich, St. Louis, MO) (Carson et al. 2002). Total protein concentrations were determined by the Bradford method. Equal amounts of crude protein homogenates were fractioned on hand‐casted 6.5%–10% SDS‐polyacrylamide gels and electrophoretically transferred to a PVDF membrane, using a Genie Blotter (IDEA Scientific, Minneapolis, MN). Membranes were stained with a Ponceau S solution in order to verify equal protein loading and transfer efficiency.

Membranes were blocked for one hour in a 5% milk in tris‐buffer saline solution with 0.1% Tween‐20 (TBS‐T). Primary antibodies for total (#9258) and phosphorylated‐ Thr183/Tyr185 (#4671) JNK, total (#8242) and phosphorylated‐Ser536 (#3033) NF‐*κ*B p65, and PPAR‐*γ* (#2425) from Cell Signaling (Danvers, MA), and Collagen I (#10423) and III (#0549) from Bioss (Woburn, MA) and Collagen VI (#182744) from Abcam (Cambridge, UK) were diluted 1:1000 in a 5% milk, TBS‐T solution followed by an hour or overnight incubation at 4 degrees Celsius. Anti‐rabbit or anti‐mouse IgG horseradish peroxidase conjugated secondary antibody (Cell Signaling, Danvers, MA) was incubated with the membranes at 1:2000 dilutions for one hour in a 5% milk, TBS‐T solution. Due to tissue size limitations, only HFD‐fed mice were analyzed for PPAR‐*γ* protein content.

An enhanced chemiluminescent substrate for detection of horseradish peroxidase (Thermo Scientific, Watham, MA) was used to visualize the antibody–antigen interaction. Autoradiography films were scanned and blots were quantified, using scientific imaging software (Image J). After completion of the western blot, all membranes were stained with Amido black, and the densitometry of each lane was calculated, using Quantity One Software (BioRad, Hercules, CA) allowing for total protein normalization as previously described (Enos et al. 2016). This method of normalization has been shown to be more accurate than typically used loading controls (Aldridge et al. 2008) and has been utilized in our group's previous publications (Enos et al. 2016; Velázquez et al. 2016).

### Statistical analyses

All data were analyzed, using commercial software (SigmaStat, SPSS, Chicago, IL). All outcomes were analyzed using a two‐way ANOVA (genotype x diet), except for PPAR*γ* western blot analysis, which was analyzed by a Two‐Tailed Student's *t*‐Test. Student–Newman–Keuls test was used for all post hoc analyses. Any data that were not normally distributed or did not display equal variance were logarithmically transformed so that these criterion were met. Statistical significance was set with an alpha value of *P* < 0.05. Data are presented as mean (±SEM).

## Results

### miR155 deficiency has no effect on body weight, but influences body composition and visceral fat accumulation in male mice

**Figure 1:**
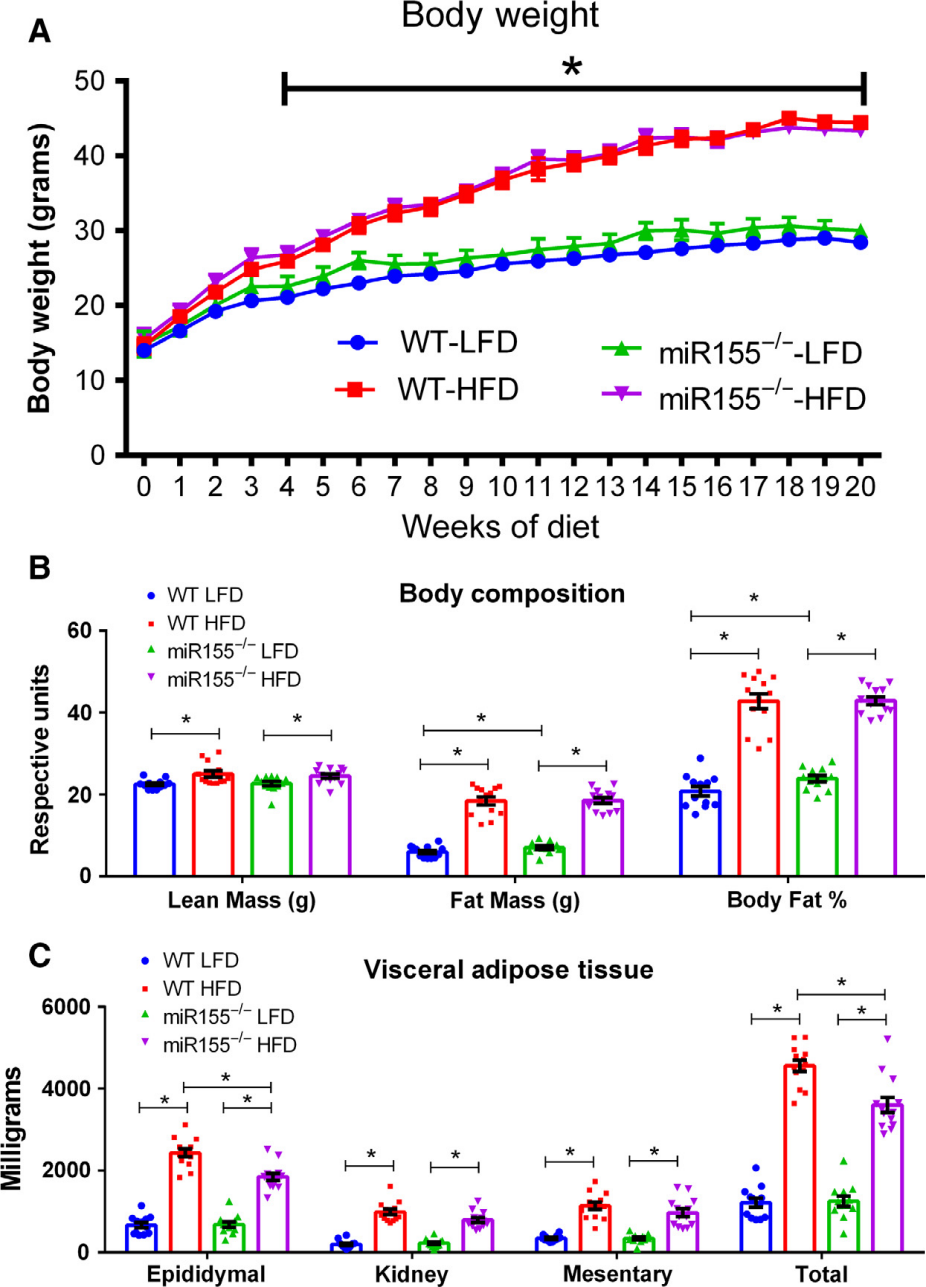
Body morphology after 20 weeks of dietary treatment is influenced by diet and genotype in miR155^−/−^ and WT male mice. (A) Body weights, (B) body composition, and (C) fat pad weights (retroperitoneal, mesentery, epididymal, total) (*n* = 13–14). *Significantly different for LFD versus high‐fat diet (HFD)‐fed mice independent of genotype (*P* < 0.05) in Figure A. For all other Figures a statistically significant difference (*P* < 0.05) is marked by an “*” and representative lines.

Body weight over the course of the dietary treatment is presented in Figure 1A. There was no effect of genotype to influence body weight, however, diet did have an effect as all HFD‐fed mice displayed a greater body weight beginning after 4 weeks of dietary treatment compared to the LFD‐fed mice (*P* < 0.05).

With respect to body composition (body fat mass (grams), lead body mass (grams), body fat %), no differences were detected in baseline measurements among the groups (data not shown). However, at the conclusion of the study, there was a significant effect of the HFD to increase lean mass, fat mass, and body fat % compared to the LFD‐fed mice (Fig. 1B, *P* < 0.05). There was also an interaction between genotype and diet within the LFD‐fed mice, as the miR155^−/−^ LFD mice presented a greater fat mass and body fat % (Fig. 1B, *P* < 0.05). Regarding visceral adipose tissue, there was an effect of the HFD to increase all fat pad weights and total visceral adipose tissue compared to the LFD‐fed mice (Fig. 1C, *P* < 0.05). Additionally, within the HFD‐fed mice, an effect of genotype was found, as the miR155^−/−^ mice displayed less epididymal and total visceral adipose tissue accrual (Fig. 1C, *P* < 0.05).

### Adipose tissue inflammation is not greatly affected by loss of miR155 in male mice

**Figure 2:**
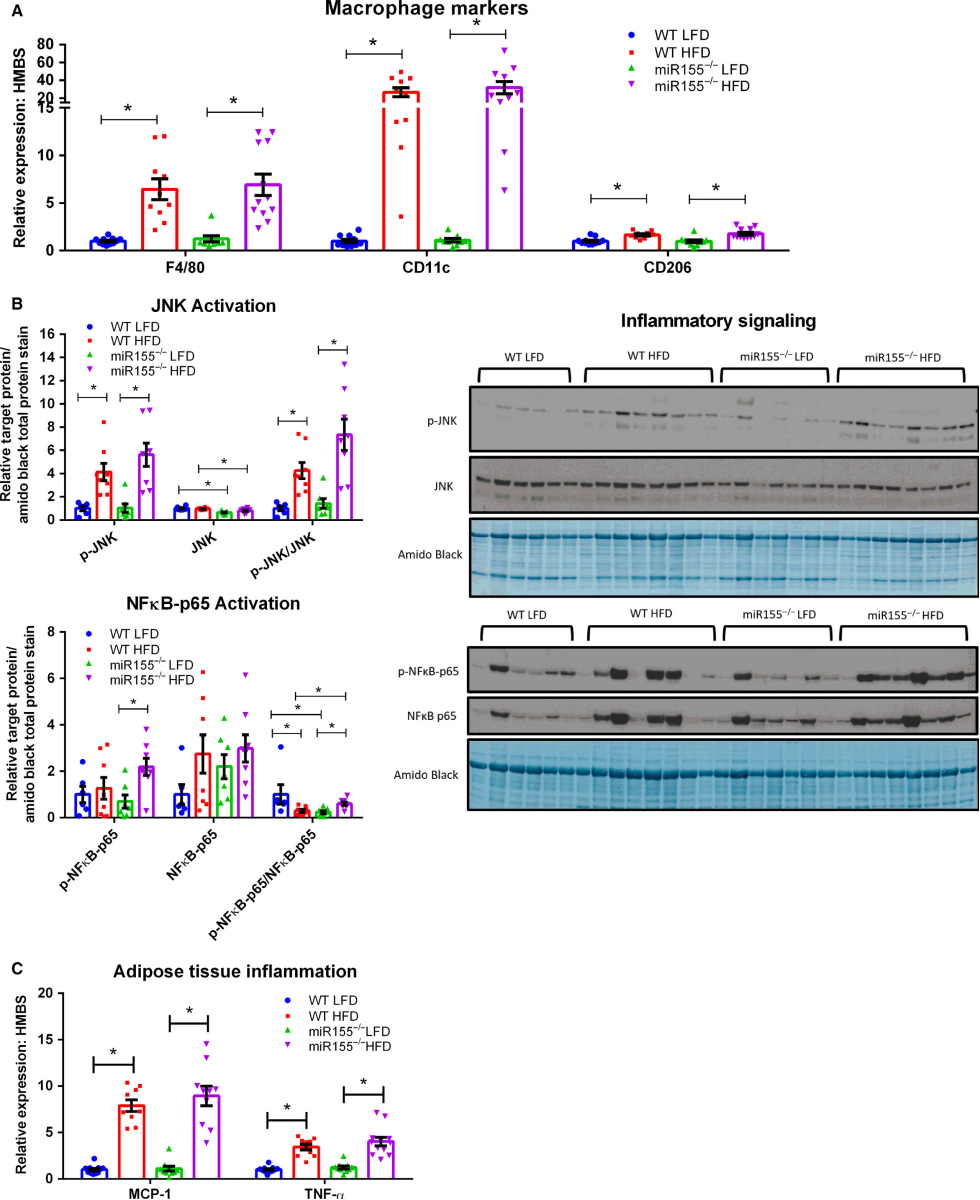
Adipose tissue inflammation is influenced by 20 weeks of dietary treatment, but not genotype in miR155^−/−^ and WT male mice. (A) Epididymal adipose tissue gene expression of macrophage markers (*n* = 10–12), (B) representative epididymal adipose tissue western blots of phosphorylated (Thr183/Tyr185), total JNK and phosphorylated:total JNK, phosphorylated NF *κ*B‐p65 (Ser536), total NF *κ*B‐p65, and phosphorylated:total NF *κ*B (*n* = 6–8), and (C) epididymal adipose tissue gene expression of inflammatory markers (MCP‐1 and TNF‐*α*) (*n* = 10–12). *Significantly different (*P* < 0.05).

Gene expression analysis of adipose tissue macrophage markers corresponding to overall (F4/80), M1 (CD11c), and M2 (CD206) macrophages is presented in Figure 2A. For each of these markers, only an effect of diet was evident, as all HFD‐fed mice displayed increased mRNA expression compared to LFD‐fed mice (*P* < 0.05). It should be noted that an attempt was made to examine adipose tissue macrophage infiltration (F4/80 immunohistochemistry) in order to corroborate the qRT‐PCR data as in our previous publications (Enos et al. 2013, 2014a, 2016). However, due to unspecific positive staining as a result of the elevated adipose tissue fibrosis exhibited by the mir155^−/−^ HFD mice, we were unable to accurately assess macrophage infiltration by immunohistochemistry.

With regard to inflammatory signaling, adipose tissue JNK and NF*κ*B activation were examined. JNK is known to play a central role in adipose tissue inflammation and insulin resistance (Hirosumi et al. 2002) and we have previously found JNK phosphorylation to be elevated in the adipose tissue of mice consuming the same HFD utilized in this experiment (Enos et al. 2013, 2014a, 2016; Cranford et al. 2016). NF*κ*B can regulate chemokine expression in inflamed adipose tissue (Tourniaire et al. 2013) and its activation has recently been shown to promote survival of adipose tissue macrophages under obese conditions (Hill et al. 2015). We found JNK activation and the p‐JNK:JNK followed a similar pattern as the macrophage markers; HFD consumption increased these outcomes relative to LFD‐consumption (Fig. 2B, *P* < 0.05). Interestingly, total JNK protein content was found to be influenced by genotype, as miR155^−/−^ mice displayed less total adipose tissue JNK regardless of diet (Fig. 2B, *P* < 0.05). No differences in total adipose tissue NF*κ*B‐p65 protein content was found among the groups (Fig. 2B). There was, however, an effect of HFD consumption within the miR155^−/−^ mice to increase adipose tissue p‐NF*κ*B‐p65 content (Fig. 2B, *P* < 0.05). Interestingly, when examining the adipose tissue p‐NF*κ*B‐p65:NF*κ*B‐p65, a genotype and diet interaction was found: both WT LFD and miR155^−/−^ HFD mice displayed a greater p‐NF*κ*B‐p65:NF*κ*B‐p65 compared to miR155^−/−^ LFD and WT HFD mice (Fig. 2B, *P* < 0.05).

Analysis of adipose tissue inflammatory markers is presented in Figure 2C. Both TNF‐*α* and MCP‐1 mRNA expression were found to be influenced by diet as consumption of the HFD increased their expression relative to LFD‐fed mice (*P* < 0.05).

### Lack of miR155 augments adipose tissue fibrosis in male mice under HFD consumption paired with a decrease in adipose tissue PPAR‐*γ* protein content

**Figure 3:**
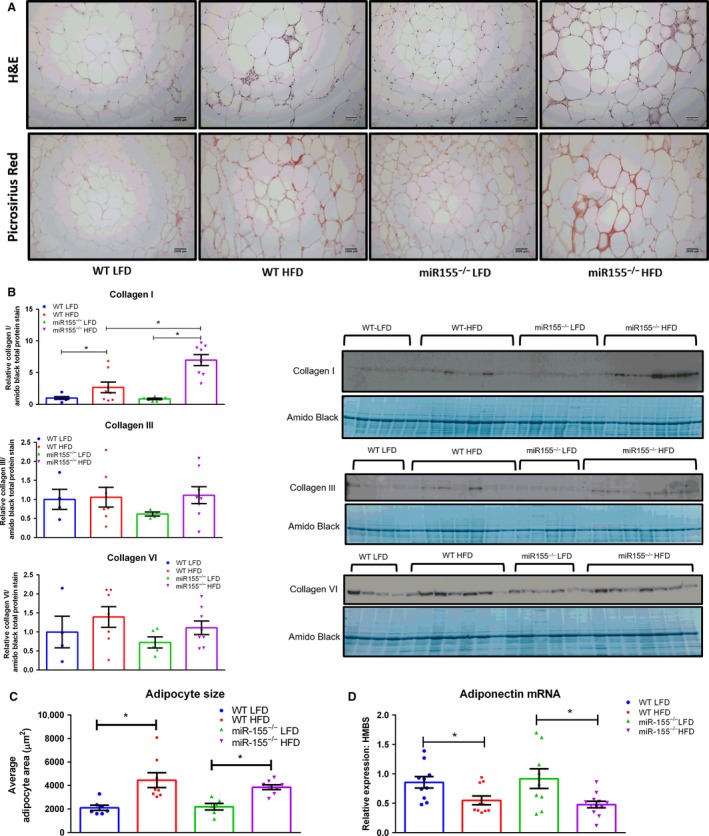
miR155 deletion increases adipose tissue fibrosis under high‐fat diet (HFD) conditions in male mice. (A) Representative images of H&E and Picrosirius Red staining in epididymal AT (20x), (B) representative western blots of Collagen I, III, and VI (*n* = 4–8), (C) average epididymal adipocyte size (*n* = 6–8), and (D) epididymal adipose tissue gene expression of adiponectin. *Significantly different (*P* < 0.05).

Representative H&E and Picrosirius Red images are presented in Figure 3A. Picrosirius Red positively stains type I and III collagen. Regardless of genotype, it was evident that consumption of the HFD increased adipose tissue fibrosis and collagen formation compared to LFD‐fed mice. Additionally, it was clear that a genotype effect existed within the HFD‐fed mice, as adipose tissue fibrosis was more pronounced in miR155^−/−^ mice.

The histological images were supported by western blot of Collagen I, as there was a HFD effect to increase Collagen I protein content relative to LFD‐fed mice as well as a genotype x diet interaction as miR155^−/−^ HFD mice exhibited increased Collagen I protein compared to WT HFD mice (Fig. 3B, *P* < 0.05). Although there was an increase (~50%) in Collagen VI in adipose tissue with HFD this did not reach statistical significance (Fig. 3B). No differences among the groups were found with respect to adipose tissue Collagen III (Fig. 3B).

With respect to average adipocyte size, there was an effect of diet on this outcome, as all HFD‐fed mice presented a larger average adipocyte area (Fig. 3C, *P* < 0.05). Adiponectin adipose tissue gene expression was also found to be influenced by diet, as all HFD‐fed mice displayed decreased adipose tissue adiponectin mRNA expression (Fig. 3D, *P* < 0.05).

**Figure 4:**
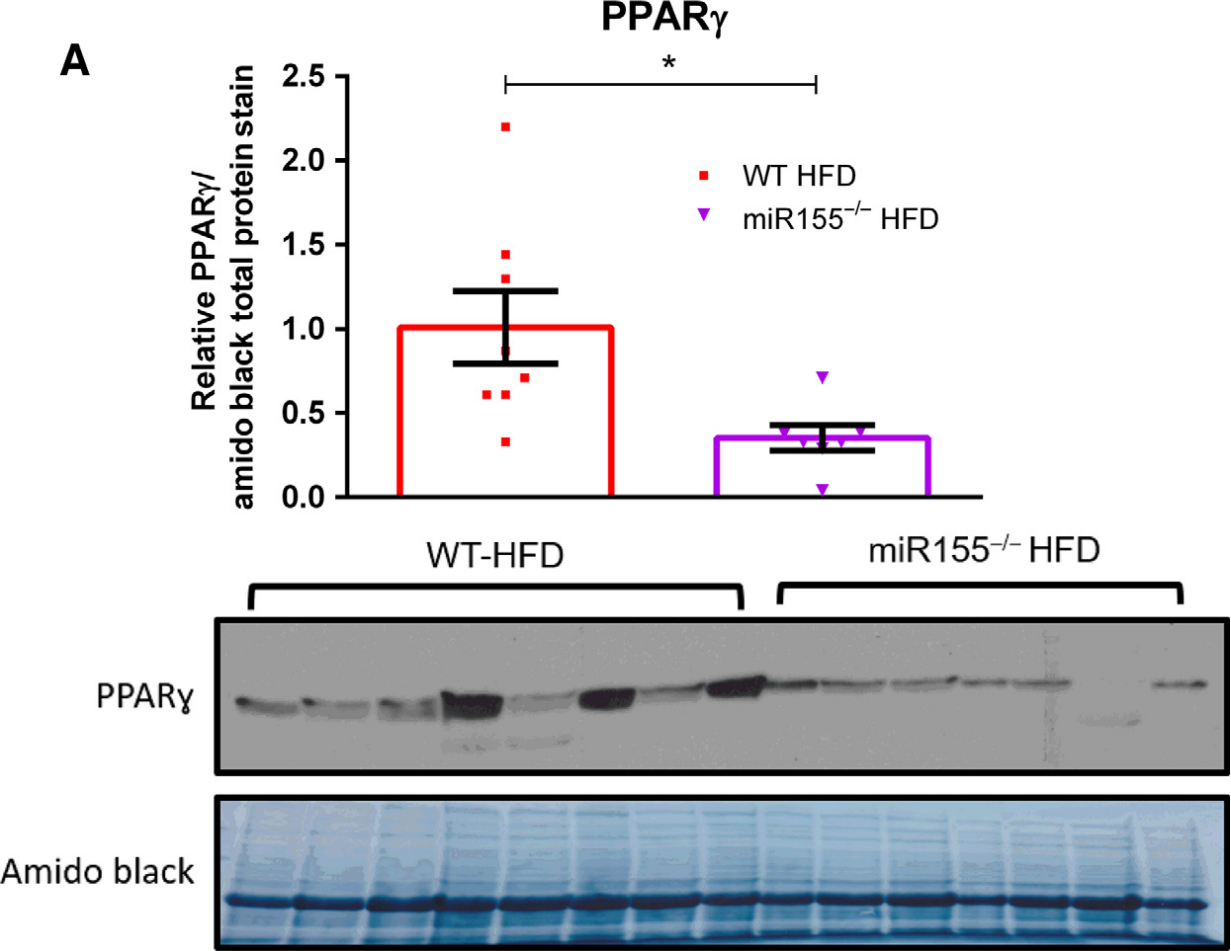
Adipose Tissue PPAR *γ* Content, a marker of adipogenesis, is decreased in miR155^−/−^ high‐fat diet (HFD)‐fed male mice. (A) Representative western blot of epididymal adipose tissue PPAR‐*γ* content (*n* = 7–8). *Significantly different (*P* < 0.05).

Protein content of PPAR‐*γ*, a marker of adipose tissue adipogenesis, was found to be significantly decreased in miR155^−/−^ HFD mice compared to WT HFD mice (Fig. 4, *P* < 0.05).

### HFD consumption influences glucose metabolism and insulin resistance

**Figure 5:**
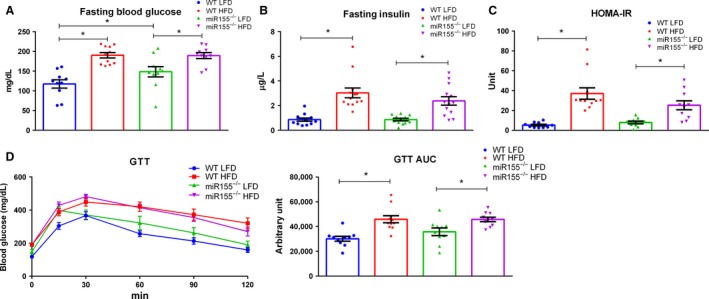
Glucose metabolism in not impacted by miR155 deletion under high‐fat diet (HFD) conditions in male mice. (A) Fasting blood glucose, (B) fasting plasma insulin, (C) HOMA‐IR, and glucose tolerance test after 19 weeks of dietary treatment (*n* = 13–14). *Significantly different (*P* < 0.05).

There was an effect of HFD to increase fasting blood glucose, fasting insulin, HOMA‐IR, and GTT AUC, compared to LFD consumption (Fig. 5A, B, C, and D, *P* < 0.05). A genotype x diet interaction was found only with respect to fasting blood glucose as miR155^−/−^ LFD mice displayed a greater fasting blood glucose concentration compared to WT counterparts (Fig. 5A, *P* < 0.05).

## Discussion

While the role of miR155 in the development of obesity remains unclear, our study was designed to assist in unraveling the role of miR155 in the development of the obese phenotype. We extend this field of research through the examination of miR155 deletion on adipose tissue inflammation in an obese setting in male mice. Utilizing an HFD‐induced obese model, we found that miR155 deletion in male mice resulted in reduced visceral adipose tissue, but did not rescue adipose tissue inflammation or impaired glucose metabolism. Surprisingly, these outcomes were evident in the presence of exacerbated adipose tissue fibrosis induced by miR155 deficiency in HFD‐fed mice.

Since miR155 can serve as a pro‐inflammatory molecule, particularly in inducing M1, pro‐inflammatory, macrophage polarization, we hypothesized that miR155 deficiency would attenuate adipose tissue inflammation ultimately resulting in improved glucose metabolism (Essandoh et al. 2016; Jablonski et al. 2016; Zhang et al. 2016). However, no such effect was found, as both WT and miR155^−/−^ mice displayed similar gene expression profiles with respect to adipose tissue macrophage polarization and inflammatory markers (MCP‐1 & TNF‐*α*), JNK activation, and impaired glucose metabolism and insulin resistance. Interestingly, these outcomes presented themselves even though miR155^−/−^ HFD mice accumulated less visceral adipose tissue, specifically with respect to the epididymal fat pad, compared to WT HFD mice.

Our findings agree with a recently published paper by Gaudet et al. (2016); deletion of miR155 in male mice resulted in less HFD‐induced visceral adipose tissue accumulation, yet WT and miR155 knockout mice has similar body weight gain and impaired glucose metabolism. However, Gaudet *et al*. did not examine adipose tissue inflammation. The finding that miR155^−/−^ HFD mice displayed less total visceral fat mass, yet displayed a similar amount of total fat, as determined by DEXA analysis, suggests that miR155 loss may have enhance the capacity for excess energy storage in subcutaneous adipose tissue depots as compared to visceral depots in male mice. This is further supported by the differences in fat mass between the miR155^−/−^ and WT LFD mice; miR155^−/−^ LFD mice had more body fat compared to the WT LFD mice, yet no differences in visceral fat were evident (similar to the finding of Virtue et al. (2016)). miR155 also has been shown to be a negative regulator of brown adipose tissue function and white adipose tissue “browning”(Chen et al. 2013). Although we did not specifically examine differences in the white adipose tissue browning it may be that miR155 deletion affected distribution of adipose tissue due to its ability to influence adipose tissue phenotype. However, further work is needed to establish this mechanism.

In order to explain the rationale behind the reduced epididymal fat pad size, yet similar degrees of inflammation and insulin resistance, we histologically examined the epididymal fat pad. Neither average adipocyte size nor adipocyte size frequency (data not shown) was found altered by miR155 loss in HFD‐fed mice. Yet, HFD‐fed miR155^−/−^ mice had accentuated fibrosis in the epididymal fat pad. Since fibrosis is characteristic of adipose tissue dysfunction and has been shown to impair metabolic processes (Sun et al. 2013), we were surprised to find similar degrees of adipose tissue inflammation and metabolic impairments between WT and miR155^−/−^ HFD‐fed mice. To further characterize the fibrosis, we performed western blots for Collagen I, III, and VI all of which have been reported to play a role in ECM remodeling in adipose issue. Collagen type I and III are more frequently observed in fibrous bundles, providing the major ECM framework, whereas collagen type VI surrounds parenchymal adipocytes having a pivotal role in ECM stability. Collagen VI is thought to be the most abundantly expressed collagen in adipose tissue and has previously been implicated as a key player in metabolic dysregulation and adipose tissue fibrosis (Khan et al. 2009). Collagen I was the only collagen found to be increased with miR155 loss. The role of adipose tissue Collagen I in regulating metabolic dysfunction has not been fully elucidated to the extent of Collagen VI. It may be that changes in Collagen VI protein content, and not solely in Collagen I protein levels, are necessary to impair metabolic processes. However, our findings should be interpreted with caution given that these proteins were assessed at only one time‐point. Given the different roles of fibrotic proteins in adipose tissue, a full evaluation of the role of miR155 on fibrosis in an obesity model would require measurement at different stages of obesity development.

Adiponectin is an insulin‐sensitizing, adipose‐tissue‐derived adipokine that can regulate adipose tissue collagen levels and protect against adipose tissue fibrosis formation (Khan et al. 2009; Crewe et al. 2017). We hypothesized that adipose tissue gene expression of adiponectin may be decreased in miR155^−/−^ HFD mice compared to WT HFD mice. We found that HFD‐feeding decreased adiponectin gene expression regardless of miR155 loss, suggesting that a change in adiponectin is not likely the regulator of the increased adipose tissue fibrosis displayed by miR155^−/−^ HFD mice.

Impairments in adipogenesis is another hypothesized mechanism to explain the elevated adipose tissue fibrosis exhibited by miR155^−/−^ HFD mice. We performed a western blot for adipose tissue PPAR‐*γ*, a master regulator of adipogenesis (Shao et al. 2016) and known target of miR155 (Karkeni et al. 2016). Interestingly, we found PPAR‐*γ* protein content to be decreased in the adipose tissue of miR155^−/−^ HFD mice compared to WT HFD mice. An impairment in adipogenesis may explain the increase in adipose tissue fibrosis displayed by miR155^−/−^ HFD mice. However, it should be noted that additional in vivo experiments, under various conditions, are necessary in order to fully comprehend miR155′s role in regulating adipogenesis. This is considering the finding that a potential decrease in adipogenesis exhibited by miR155^−/−^ mice under HFD conditions would be contradictory to in vitro data suggesting that miR155 is an inhibitor of adipogenesis. For example, Liu et al. (2011) showed that miR155 may serve as a potent inhibitor of adipogenesis in vitro, and Gaudet et al. (2016) found that miR155^−/−^ preadipocytes differentiated more readily than WT preadipocytes. Thus, in order to explain our finding, we speculate that miR155 may influence the adipogeneic capacity of various fat pads differently (e.g., miR155 deletion may favor subcutaneous adipose tissue adipogenesis over gonadal fat pad adipogenesis). However, more research is needed in order to support this potential hypothesis.

It is evident that more studies examining the role of miR155 on obesity development are necessary given the discrepancies between the findings of several investigations, including our own. For example, both our investigation and that of Gaudet et al. (2016) found that miR155^−/−^ mice subjected to a HFD resulted in decreased visceral adipose tissue accumulation compared to HFD‐fed WT mice. Virtue et al. (2016), however, found no such effect as both miR155^−/−^ and WT HFD‐fed mice displayed a similar amount of gonadal fat accretion. Furthermore, our results, as well as those of Gaudet et al. (2016)*,* suggest that miR155 deficiency in a male mouse model exposed to an HFD results in similar degree of impaired glucose metabolism and insulin resistance as WT HFD‐fed mice. Lin et al. (2016), however, found that miR155 deletion in male mice resulted in poorer glucose metabolism and greater insulin resistance than WT controls.

Sex differences may also be a factor that impacts the miR155 deficient phenotype exposed to an HFD. For example, Gaudet et al. (2016) found miR155 deficiency in female mice to be effective at preventing diet‐induced obesity as characterized by decreased body weight gain, increased expression of adipogenic genes, blunting of adipose tissue inflammation, and improved insulin sensitivity. Virtue et al. (2016), on the other hand, found that miR155 deficiency in female mice led to a similar body weight gain and body fat accumulation as WT‐HFD‐fed mice and Lin et al. (2016) discovered that miR155^−/−^ female mice exposed to an HFD actually displayed impaired glucose metabolism and insulin signaling compared to WT HFD‐fed controls. There are many factors which may account for the discrepancies between studies. For example, the diet utilized in this study was designed to be similar to the standard American diet and has been previously used in other investigations (Enos et al. 2013, 2014a,b, 2016). This diet is different than the majority of other HFDs utilized in obesity investigations as typical HFD diets tend to be much higher in total (approximately 60% in some cases) and saturated fat (up to 25%). As such, it is possible that the diet used in our investigation may have affected the outcomes differently than what others have found. Additionally, other factors such as duration of feeding, potential differences in the gut microbiome and the fact that WT littermate controls were not utilized may also have impacted the results. It is evident that more studies need to be performed in order to fully comprehend the role of miR155 in obesity development.

In conclusion, this study suggests that, in male mice, miR155 deletion in an HFD model of diet‐induced obesity reduces visceral adipose tissue while exacerbating adipose tissue fibrosis. Interestingly, miR155 loss had no effect on adipose tissue inflammation or glucose metabolism. This study serves to extend our understanding of the complex role of miR155 in obesity. However, our study points to the need for additional important future investigations examining sex‐specific effects, differences in HFD duration, and tissue‐specific changes to fully comprehend the extent to which miR155 regulates the development of the obese phenotype.

## Conflict of Interest

The authors declare they have no Conflict of interest.
